# A Hard Case

**Published:** 1888-05-12

**Authors:** 


### "A HARD CASE."

IF you were to write to the secretary sister, St. Peter's Home, Kilburn, N.W., I am sure they would take in the case mentioned, having a ladies' ward, about 10*s*. 6*d*. per week extra, of course; and also the case could be moved to London in an ambulance by train, never moving patient, as I know it has been done, and the sister would give all information about expense. In the end would be cheaper; if not, I think some one would help case at 10*s*. per week to nurse it. A. B.

